# Prevalence and factors associated with food insecurity in eight high-altitude cities in Peru during the second wave of the COVID-19 pandemic: a retrospective, cross-sectional study

**DOI:** 10.1186/s12889-022-14372-6

**Published:** 2022-10-25

**Authors:** J. Pierre Zila-Velasque, Pamela Grados-Espinoza, Katherine Quispe-Chura, Christopher G. Valdiviezo-Morales, Cristian Diaz-Vélez, Mario J. Valladares-Garrido

**Affiliations:** 1 School of Medicine, Universidad Nacional Daniel Alcides Carrión, Pasco, Peru; 2 Red Latinoamericana de Medicina en La Altitud E Investigación (REDLAMAI), Pasco, Peru; 3 School of Medicine, Universidad Nacional del Altiplano, Puno, Peru; 4 School of Medicine, Universidad Nacional de Piura, Piura, Peru; 5 Scientific Society of Medical Students, Universidad Nacional de Piura, Piura, Peru; 6 School of Medicine, Universidad Privada Antenor Orrego, Trujillo, Peru; 7 Instituto de Evaluación de Tecnologías en Salud e Investigación – IETSI, EsSalud, Lima, Peru; 8 Universidad Continental, Lima, Peru; 9 Oficina de Epidemiología, Hospital Regional Lambayeque, Chiclayo, Peru

**Keywords:** High-altitude cities, COVID-19, Food security, Mental health, Public health, Peru

## Abstract

**Background:**

Food insecurity has increased during the COVID-19 pandemic, affecting an estimated 260 million people. However, little evidence is available on how pandemic-related characteristics influence food security in a high-altitude population. The objective of this study was to assess factors associated with food insecurity in high-altitude Peruvian cities during the second epidemic wave of COVID-19.

**Methods:**

A retrospective, cross-sectional study was conducted in eight Peruvian cities over 1,500 m above sea level. An online survey measuring food security, presence of anxiety & depressive symptoms, sleep quality, post-traumatic stress disorder (PTSD), resilience, and sociodemographic characteristics was disseminated through social networks between December 2020 and February 2021. Generalized linear models were used to identify an association between the study variables.

**Results:**

Of 700 participants, the median age was 23 years, and more than half were female (56.7%). The prevalence of food insecurity was 37.1%. Anxiety symptoms, depressive symptoms, and PTSD were present in 72.7%, 64.1%, and 15% of respondents, respectively. The prevalence of food insecurity was higher in people with fair (PR: 1.60, 95% CI: 1.23–2.07) and very bad perception of their health (PR: 4.06, 95% CI: 2.63–6.26), individuals seeking mental health support (PR: 1.42, 95% CI: 1.25–1.62), and in those who lost their job due to the pandemic (PR: 1.82, 95% CI: 1.62–2.04). Having moderate (PR: 1.52, 95% CI: 1.26–1.83) and moderate to severe depressive symptoms (PR: 1.58, 95% CI: 1.11–2.27) also increased the prevalence of food insecurity.

**Conclusion:**

During the pandemic, the prevalence of food insecurity has increased in the Peruvian high-altitude population, revealing the need for preventive strategies. Identification of pandemic-related characteristics that influence food insecurity can guide interventions in at-risk individuals and reduce the long-term impact of this problem on overall health and quality of life.

## Introduction

In Peru, restrictive measures, such as mandatory quarantine, were imposed to mitigate the impact of the COVID-19 pandemic, which limited access to food, medicines, and essential services [1]. This has led to a major impact on economic activities [2] and a high frequency of food insecurity (75.5%) [3, 4]. In Latin America and the Caribbean, before COVID-19, it was estimated that 205 million people experienced food insecurity [5]. In 2020, this problem increased, affecting around 260 million people [6]. Studies conducted in the context of the pandemic found a frequency of food insecurity in Peru of around 23.2–83.9% [1, 7, 8]. A study conducted before COVID-19 in one district of Ayacucho (southern Peru) found 60.9% of food insecurity in families with at least one child under 12 years of age [7].

The impact of the pandemic has also extended to the mental health field, influencing the development of anxiety and depression as a result of confinement [9]. This situation has caused higher economic damage to vulnerable populations, such as those with low economic resources, increased by the social isolation measures that prevented daily subsistence activities [10]. Food insecurity is associated with mental health disorders [11, 12].

Relevant factors associated with food insecurity during the COVID-19 pandemic include female sex [4, 8], low educational level [13], poverty [14], anxiety [11, 14], and depression [10, 11]. The consequences of food insecurity during the pandemic are especially important for mental health, as this problem can be more serious for families, particularly those with children, than in other situations [11]. This would be explained by the primary need to provide food, which leads to constant psychological distress. However, significant characteristics related to mental health remain under-assessed, such as post-traumatic stress disorder (PTSD), search for mental health support, loss of employment, and general health perception. These characteristics may limit the ability to overcome the impact of the pandemic on economic and food security.

Studies conducted in Peru on the factors influencing food insecurity during the COVID-19 pandemic are scarce and related to the first epidemic wave [1, 4, 6]. In addition, information in high-altitude cities, including a unique geographical population, is poorly reported before this context [7]. Therefore, this study aimed to evaluate the factors associated with food insecurity in eight high-altitude cities during the second wave of the COVID-19 epidemic in Peru. This information would allow a better understanding of how the frequency of food insecurity has differed in the Peruvian population to recognize at-risk groups. The study will contribute to the literature by explaining underexplored characteristics that might influence the occurrence of food insecurity, showing possible variation in its prevalence compared to other populations and epidemic waves, and providing evidence for developing strategies to prevent this public health problem.

To this end, the following research questions were addressed: 1) What is the prevalence of food insecurity in high-altitude Peruvian cities during the second epidemic wave of COVID-19? 2) How does the prevalence of food insecurity vary by participant characteristics? 3) Are experiences considered to be consequences of the pandemic (loss of employment, health perception, search for mental health support, PTSD, diagnosis of COVID-19, family members with COVID-19) associated with the development of food insecurity? In light of these questions, we hypothesized that 1) there is a high prevalence of food insecurity in high-altitude cities during the second epidemic wave, 2) the prevalence of food insecurity varies according to the characteristics of the participants, especially those related to mental health, and 3) pandemic-related experiences are associated with the development of food insecurity.

## Methods

### Study design

A retrospective study was conducted using data from a previous cross-sectional survey assessing the association of resilience with mental health outcomes in eight Peruvian high-altitude cities during the second epidemic wave of COVID-19. The study followed an analytical approach that aimed to compare the prevalence of food insecurity among people exposed and not exposed to the potential risk factor. The cities were over 1,500 m above sea level and included Apurímac, Ayacucho, Cajamarca, Cuzco, Huancavelica, Junín, Pasco, and Puno.

### Study population and sample

The study population consisted of people over 18 years of age residing in eight high-altitude cities (population size of 6 109 058 inhabitants [15]). For the primary study, a sample size of 450 individuals was estimated based on a 95% confidence interval, a statistical power of 99%, and a resilience prevalence ratio of 1.5. To this number was added 20% incomplete responses (*n* = 90) and 20% refusals (*n* = 90). The final sample size was 630 individuals.

### Procedure

**Fig. 1:**
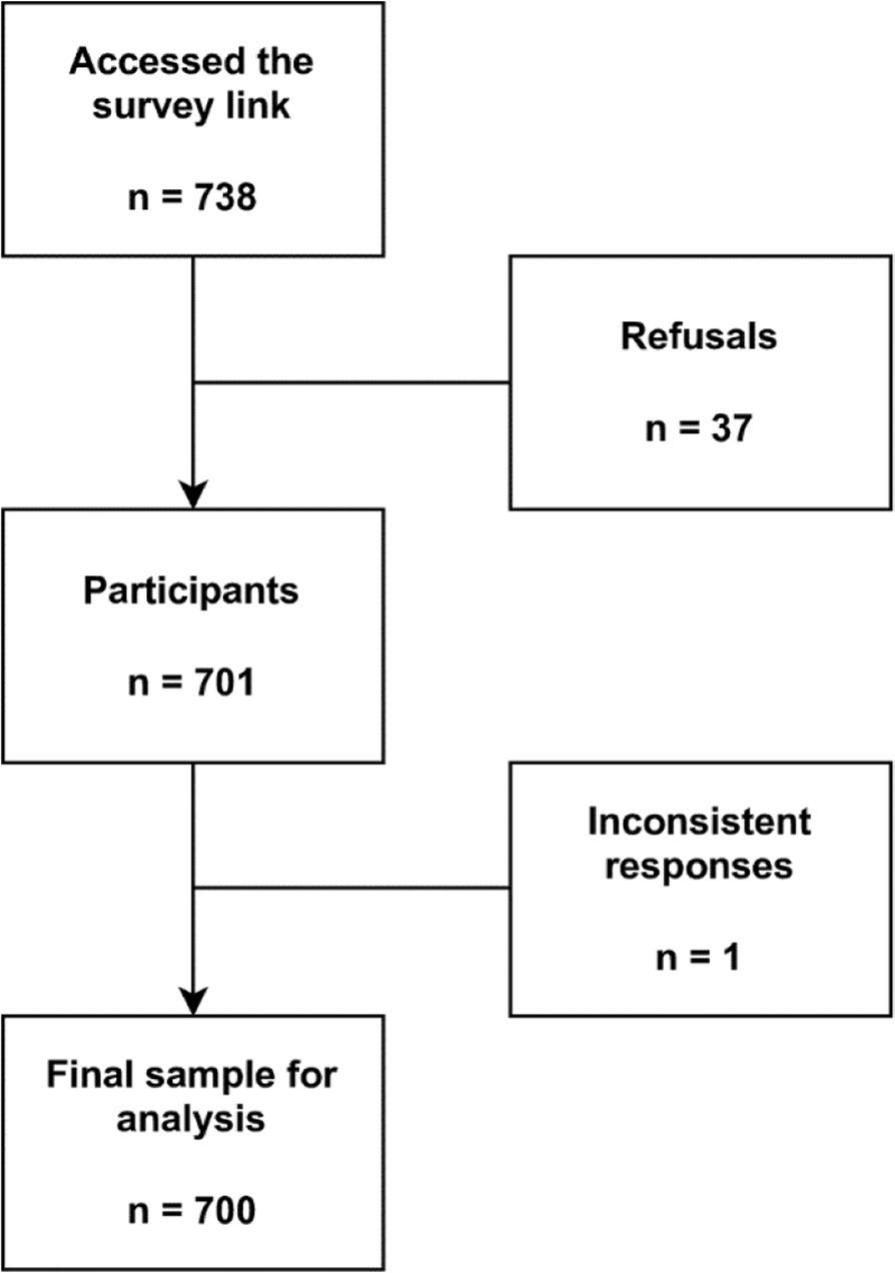
Participant selection flowchart

A list of validated questionnaires (indicated in the *Measures* section) was compiled to develop a virtual survey designed with Google® Forms. The survey was shared between December 20, 2020, and February 28, 2021 (the initial period of the second epidemic wave in Peru) through social networks (i.e., WhatsApp®, Instagram®, Facebook®, and Telegram®). The authors were supported by collaborators residing in the selected high-altitude cities, who also disseminated the survey using a snowball sampling method. To prevent bot responses and multiple responses from one individual, we asked each participant to log in with their Gmail® account before starting the survey (the email address was not recorded in the survey). Potential participants who entered the survey had two initial questions on the first page that asked if the individual was over 18 years of age and lived in any selected high-altitude city. Those who met the initial selection criteria moved to a new section where they were shown informed consent and the option to voluntarily participate in the study. The time to complete the survey was indicated as approximately 10 min. No personal data were requested, and the answers provided in the research remained anonymous and confidential. A total of 701 participants accepted to participate and completed the survey, resulting in a response rate of 94.9%. Once the required number of participants had been completed, the database was downloaded and reviewed for inconsistent or duplicate values. Only one observation was excluded due to incorrect data. The participant selection process is detailed in Fig. 1.

The characteristics of the sample (*n* = 700) are shown in Table 1. The mean age was 23 years, most participants were female (56%, *n* = 394), with incomplete higher education (44%, *n* = 310), and the main occupation reported was that of university/technical students (65%, *n* = 455).

**Table 1:** Sample characteristics (*n* = 700)

Characteristics	n (%)
**Age (years)**^**a**^	23 (18–70)
**Sex**	
Female	394 (56.7)
Male	301 (43.3)
**Education level**	
Without formal education	1 (0.1)
Incomplete primary	3 (0.4)
Complete primary	4 (0.6)
Incomplete secondary	24 (3.4)
Complete secondary	139 (19.9)
Incomplete higher education	310 (44.3)
Complete higher education	164 (23.4)
Postgraduate	55 (7.9)
**Occupation**	
Housewife/Retired	30 (4.3)
Public/Private institutions employees	162 (23.1)
University/Technical students	455 (65.0)
Others	18 (2.6)
**Religion**	
Catholic	447 (63.9)
Evangelical	91 (13.0)
Others	72 (10.3)
None	90 (12.9)
**Comorbidity history**	
No	595 (85.0)
Yes	105 (15.0)
**Specific comorbidity**	
None	596 (85.1)
Asthma	12 (1.7)
Diabetes	1 (0.1)
Hypertension	10 (1.4)
Obesity	13 (1.9)
Others	64 (9.1)
**History of mental health disorder**	
No	637 (91.0)
Yes	63 (9.0)
**Specific mental health disorder**	
None	638 (91.1)
Anxiety symptoms	17 (2.4)
Depressive symptoms	14 (2.0)
Bipolar disorder	4 (0.6)
Obsessive compulsive disorder	8 (1.1)
Post-traumatic disorder	5 (0.7)
Others	14 (2.0)

^a^Median (min–max value)

### Measures

*Food insecurity* was measured with the Household Food Insecurity Access Scale (HFIAS). The HFIAS was developed by the US Agency for International Development [16] and includes nine items, corresponding to questions about food consumed in the last four weeks. Participants are asked about the quality and insufficient consumption of food, physical consequences, and anxiety secondary to food insecurity. Responses are classified as follows: food security (item 1), mild food insecurity (item 2 to 4), moderate food insecurity (item 5 or 6), and severe food insecurity (item 6 to 9) [17]. Mild food insecurity presents with scores of 2–3 on item one, 1–3 on item two, or one on item three or four. Moderate food insecurity presents with scores of 2–3 on items three or four, or 1–2 on items five or six. Severe food insecurity presents with scores of three on items five or six, or 1–3 on items seven, eight, and nine. The Spanish version of the instrument has been validated in the Peruvian population [18]. The HFIAS has shown high internal consistency (α = 0.74) [19]. For this study, Cronbach's alpha coefficient was 0.87.

*The presence of anxiety symptoms* was measured with the Generalized Anxiety Disorder-7 scale (GAD-7), a unidimensional self-administered instrument designed to evaluate the presence of GAD symptoms [20]. A cut-off point was identified for adequate sensitivity (89%) and specificity (82%) [21]. This scale contains seven items, with scores ranging from 0 (not at all) to 3 (almost every day). The overall score ranges from 0 to 21. The mean scores were categorized as absence of anxiety symptoms (score of 0–4 points), mild anxiety symptoms (5–9 points), moderate anxiety symptoms (10–14 points), and severe anxiety symptoms (15–21 points). The Spanish version of the instrument has been previously validated in the Peruvian population, showing high internal consistency with a Cronbach's alpha of 0.89 [22]. For the present study, this coefficient was 0.93.

*The presence of depressive symptoms* was measured with the Patient Health Questionnaire-9 (PHQ-9), a psychometrically reliable instrument to screen for depression, which was validated to use in the primary health care system in Peru [23]. The PHQ-9 has nine items that assess the presence of depressive symptoms (corresponding to DSM-IV criteria), present in the last two weeks. Each item follows a three-point Likert scale, from 0 = "never", 1 = "some days", 2 = "more than half of the days" to 3 = "almost every day" [24]. The mean scores of depressive symptoms were categorized as minimal (0–4 points), mild (5–9 points), moderate (10–14 points), moderate to severe (15–19 points), and severe (20–27 points). The instrument shows optimal values of sensitivity (88%) and specificity (92%) and has an acceptable internal consistency with a Cronbach's alpha coefficient of 0.84 [24]. For this study, the coefficient was 0.93.

*Sleep quality* was measured with the Oviedo Sleep Questionnaire (OSQ), a self-administered instrument that helps diagnose sleep disorders such as insomnia and hypersomnia, according to DSM-IV and ICD-10 criteria. The OSQ has 15 items, 13 of which are grouped into three scales: 1) subjective sleep satisfaction, 2) insomnia, and 3) hypersomnia. The score ranges from 9 to 45 points (the higher the score, the higher the severity). All items of the instrument follow a Likert-type response scale. Subjective sleep satisfaction subscale scores range from one to seven points. The insomnia subscale scores range from 9 to 45 points. Hypersomnia subscale scores range from three to 15 points [25]. The OSQ has been validated in the Spanish population [26]. The internal consistency of the insomnia subscale was 0.91 and that of the hypersomnia subscale was 0.88. The internal consistency of the overall OSQ scale was high with a Cronbach's alpha coefficient of 0.90 [26]. For the present study, this coefficient was 0.81.

*Post-traumatic stress disorder* was measured with the PTSD Checklist – Civilian Version (PCL-C). It includes 17 items, corresponding to the set of symptoms identified in the DSM-IV-TR for criteria B, C, and D (intrusive re-experiencing, avoidance, and hyperactivity, respectively). Through the instrument instructions, respondents are asked to indicate how much "discomfort" each of the 17 symptoms has caused them during the past month, using a five-point Likert scale from 1 = "no discomfort”, 2 = "a little", 3 = "moderately", 4 = "a lot", to 5 = "too much". The overall score ranges from 17 to 85 points. A score of 44 or higher was considered indicative of PTSD [27]. The Spanish version of the instrument has been validated in the Peruvian population, showing high internal consistency (α = 0.90) [28]. For this study, Cronbach’s alpha coefficient was 0.96.

*Resilience* was measured with the 10-item Connor-Davidson Resilience Scale (CD-RISC). The 10-item CD-RISC uses a five-point Likert scale with a score from 0 to 4, with a higher score suggesting higher resilience. The Spanish version of the instrument has been validated in the Peruvian population and has good psychometric properties with a Cronbach's alpha of 0.85 [29]. For the present study, the coefficient was 0.96.

*Sociodemographic data* included age (continuous and categorized as young [18–29 years], adult [30–59 years], and older adult [60 + years]), sex, marital status, religion, comorbidities (asthma, diabetes, hypertension, obesity, and others), educational level, self-perceived health (five-point Likert scale responses from 0 = Very bad to 4 = Very good), and time at home (1 to 6 h, 7 to 12 h, and 13 to 24 h).

### Statistical analysis

Three statistical methods were applied to address each of the research questions. For the first research question, a descriptive analysis was performed including all participant characteristics, which were reported with frequencies and percentages. In the case of all continuous variables, the median and minimum–maximum ranges were reported after confirming the non-normality of the data distribution using histograms and the Shapiro–Wilk test.

For the second research question, a bivariate analysis was performed using the chi-square test to determine differences in the prevalence of food insecurity according to participant characteristics. In the case of continuous variables, the Mann–Whitney U test was used due to the non-normal distribution of the data to identify differences between the medians of the non-exposed and exposed groups. Differences were considered statistically significant if *p*-values were less than 0.05.

For the third research question, a multivariate analysis was performed using generalized linear models (GLM) with Poisson distribution, log-link function, robust variance, and places of residence as clusters. Prevalence ratios (PR) with 95% confidence intervals (95% CI) were estimated. This type of model was used because it allows binary outcomes to be evaluated. In addition, it provides a more interpretable measure of association and is appropriate when the frequency of the outcome is low (the event is rare) [30]. The study followed an exploratory approach that included two steps. Initially, a simple regression model was used to estimate the association of participants' characteristics with food insecurity. Then, variables that reached statistical significance (*p*-value < 0.05) were included in the final multiple regression model.

The data were organized in a spreadsheet using Microsoft Windows Excel® and then imported and analyzed in Stata® 16.1 (College Station, TX: StataCorp LL).

## Results

Characteristics related to the pandemic are shown in Table 2. A previous diagnosis of COVID-19 was reported by 12.7% (*n* = 89) of respondents. Anxiety symptoms were experienced by 72.7% (*n* = 509), depressive symptoms by 64.1% (*n* = 449), and PTSD by 15% (*n* = 106). A good perception of health represented approximately half of the participants (55.8%, *n* = 391), 53.4% (*n* = 374) were not working at the time of enrollment, and 14.2% (*n* = 100) reported having lost their job because of the pandemic.

**Table 2:** Characteristics related to the COVID-19 pandemic

Characteristics	n (%)
**Time at home**	
13 to 24 h	508 (72.6)
7 to 12 h	140 (20)
1 to 6 h	52 (7.4)
**Health perception**	
Very good	85 (12.1)
Good	391 (55.8)
Fair	200 (28.6)
Bad	21 (3.0)
Very bad	3 (0.4)
**COVID-19 diagnosis**	
No	611 (87.3)
Yes	89 (12.7)
**Isolation measures taken**	
No	106 (15.1)
Yes	594 (84.9)
**Perception of the severity of the COVID-19 pandemic**	
Very serious	281 (40.1)
Serious	312 (44.6)
Neutral	68 (9.7)
Overestimated	31 (4.4)
Really overestimated	8 (1.1)
**Degree of confidence in the government to manage the pandemic**	
Much trust	30 (4.3)
Some trust	306 (43.7)
Nor trust either distrust	172 (24.6)
Some distrust	117 (16.7)
Much distrust	75 (10.7)
**Family members with COVID-19**	
No	333 (47.6)
Yes	367 (52.4)
**Family member deceased due to COVID-19**	
No	557 (79.6)
Yes	143 (20.4)
**Search for mental health support**	
No	612 (87.4)
Yes	88 (12.6)
**Loss of employment due to the COVID-19 pandemic**	
Did not work	374 (53.4)
Did not lose their job	226 (32.2)
Did lose their job	100 (14.3)
**Anxiety symptoms**	
No	191 (27.3)
Yes	509 (72.7)
**Severity of anxiety symptoms**	
Absence	191 (27.3)
Mild	269 (38.4)
Moderate	152 (21.7)
Severe	88 (12.6)
**Depressive symptoms**	
No	251 (35.9)
Yes	449 (64.1)
**Severity of depressive symptoms**	
Minimum	251 (35.9)
Mild	209 (29.9)
Moderate	124 (17.7)
Moderate-severe	68 (9.7)
Severe	48 (6.9)
**Post-traumatic stress disorder**	
No	594 (85)
Yes	106 (15)
**Resilience**^**a**^	24 (13–31)
**Insomnia**^**a**^	17 (12–22)
**Hypersomnia**^**a**^	6 (4–8)
**Food insecurity**	
Food security	440 (62.9)
Mild food insecurity	123 (17.6)
Moderate food insecurity	72 (10.3)
Severe food insecurity	65 (9.3)

^a^Median (min–max value)

**Fig. 2:**
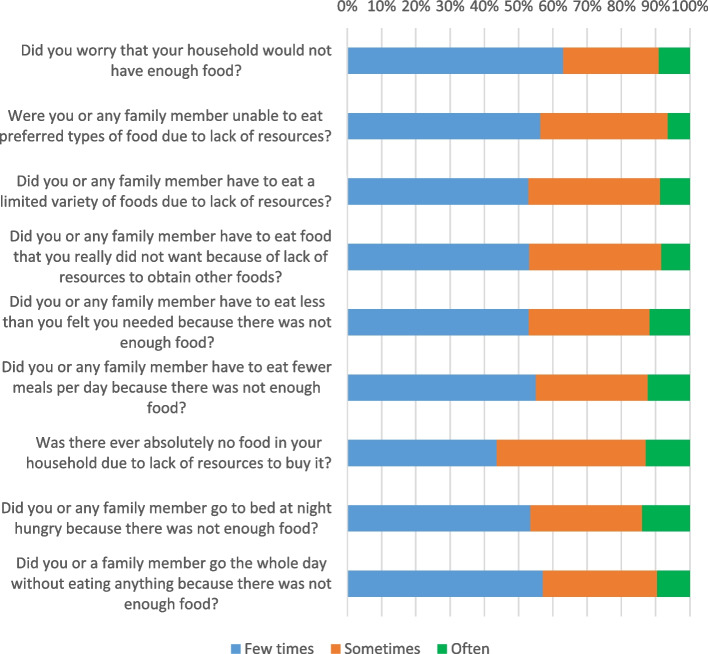
Household food insecurity access scale (HFIAS)

The prevalence of food insecurity was 37.1% (*n* = 260; 95% CI: 12.57%-18.02%). Mild food insecurity was present in 17.5% (*n* = 125), moderate food insecurity in 10.2% (*n* = 72), and severe food insecurity in 9.2% (*n* = 65) (Table 2). The HFIAS item scores are shown in Fig. 2.

The bivariate analysis (Table 3) showed significant differences regarding food insecurity prevalence according to the job (*p* = 0.019), health perception (*p* < 0.001), previous mental health disease history (*p* = 0.009), seeking mental health support (*p* = 0.002), and whether there was a loss of employment due to the pandemic (*p* < 0.001). In addition, we found a significant difference in mental health outcomes, anxiety symptoms (*p* < 0.001), depressive symptoms (*p* < 0.001), PTSD (*p* < 0.001), insomnia (*p* < 0.001), and hypersomnia (*p* < 0.001).

**Table 3:** Characteristics associated with food insecurity in bivariate analysis

Characteristics	Food insecurity		
	**No**	**Yes**	**p**
	**N (%)**	**N (%)**	
**Age group**			0.376*
Young	320 (62)	197 (38)	
Adult/Older adult	120 (66)	63 (34)	
**Sex**			0.102*
Male	200 (66)	101 (34)	
Female	238 (60)	156 (40)	
**Education level**			0.141*
Non-superior	106 (62)	65 (38)	
Incomplete higher education	185 (60)	125 (40)	
Complete higher education/postgraduate	149 (68)	70 (32)	
**Occupation**			0.019*
No	291 (60)	194 (40)	
Yes	149 (69)	66 (31)	
**Religion**			0.548*
No	54 (60)	36 (40)	
Yes	386 (63)	224 (37)	
**Comorbidity history**			0.125*
Yes	381 (64)	214 (36)	
No	59 (56)	46 (44)	
**COVID-19 diagnosis**			0.246*
No	389 (64)	222 (36)	
Yes	51 (57)	38 (43)	
**Time at home**			0.531*
13–24 h	321 (63)	187 (37)	
7–12 h	90 (64)	50 (36)	
1–6 h	29 (56)	23 (44)	
**Health perception**			0.001*
Bad/Very bad	12 (50)	12 (50)	
Fair	106 (53)	94 (47)	
Good/Very good	322 (68)	154 (32)	
**Isolation measures taken**			0.891*
Yes	374 (63)	220 (37)	
No	66 (62)	40 (38)	
**COVID-19 pandemic severity perception**			0.669*
Neutral	44 (65)	24 (35)	
Very serious/serious	374 (63)	219 (37)	
Overestimated/Really overestimated	22 (56)	17 (44)	
**Previous history of mental health disorder**			0.009*
No	410 (64)	227 (36)	
Yes	30 (48)	33 (52)	
**Family members with COVID-19**			0.174*
No	218 (65)	115 (35)	
Yes	222 (60)	145 (40)	
**Family member deceased due to COVID-19**			0.182*
No	357 (64)	200 (36)	
Yes	83 (58)	60 (42)	
**Search for mental health support**			0.002*
No	398 (65)	214 (35)	
Yes	42 (48)	46 (52)	
**Loss of employment due to the COVID-19 pandemic**			< 0.001*
Did not work	243 (65)	131 (35)	
Did not lose their job	157 (69)	69 (31)	
Did lose their job	40 (40)	60 (60)	
**Anxiety symptoms**			< 0.001*
No	142 (74)	49 (26)	
Mild	173 (64)	96 (36)	
Moderate	77 (51)	75 (49)	
Severe	48 (55)	40 (45)	
**Depressive symptoms**			< 0.001*
Minimum	186 (74)	65 (26)	
Mild	130 (62)	79 (38)	
Moderate	69 (56)	55 (44)	
Moderate to severe	30 (44)	38 (56)	
Severe	25 (52)	23 (48)	
**Post-traumatic stress disorder**			< 0.001*
No	392 (66)	202 (34)	
Yes	48 (45)	58 (55)	
Insomnia***	16 (11–21)	19 (15–24)	< 0.001**
Hypersomnia***	6 (4–8)	6 (5–9)	< 0.001**
Resilience***	25 (13–31)	24 (13–30)	0.381**

^*^P-value calculated with the chi-squared test

^**^P-value calculated with the Mann–Whitney U test

^***^Median (min–max value)

The multivariate analysis showed that food insecurity was associated with loss of employment during the pandemic (PR: 1.82, 95% CI: 1.62–2.04) and fair (PR: 1.60, 95% CI: 1.23–2.07) and very bad perception of health (PR: 4.06, 95% CI: 2.63–6.26). In addition, food insecurity was associated with the search for mental health support (PR: 1.42, 95% CI: 1.25–1.62) as well as moderate (PR: 1.52, 95% CI: 1.26–1.83) and moderate to severe depressive symptoms (PR: 1.58, 95% CI: 1.11–2.27) (Table 4).

**Table 4:** Characteristics associated with food insecurity in multivariate analysis

Characteristics	Food insecurity					
	**Simple regression**			**Multiple regression**		
	**PR**	**95% CI**	p*	**PR**	**95% CI**	p*
**Sex**						
Male	Ref			Ref		
Female	1.17	1.02- 1.35	0.020	1.06	0.94- 1.19	0.280
**Adult/Older adult**						
No	Ref					
Yes	0.90	0.71- 1.14	0.395			
**Education level**						
Others	Ref			Ref		
Incomplete higher education/course	1.06	0.92- 1.21	0.394	1.02	0.91- 1.16	0.644
Complete higher education/postgraduate	0.84	0.72- 0.98	0.029	0.84	0.68- 1.04	0.122
**Currently working**						
No	Ref					
Yes	0.76	0.62- 0.94	0.011			
**Religion**						
No	Ref					
Yes	0.91	0.74- 1.12	0.416			
**Previous comorbidity**						
No	Ref			Ref		
Yes	1.21	1.14- 1.29	< 0.001	1.03	0.92- 1.15	0.562
**COVID-19 diagnosis**						
No	Ref					
Yes	1.17	0.82- 1.68	0.377			
**Time at home**						
13–24 h	Ref					
7–12 h	0.97	0.88- 1.06	0.532			
1–6 h	1.20	0.78- 1.83	0.393			
**Health perception**						
Very good	Ref			Ref		
Good	1.54	1.13- 2.10	0.006	1.31	0.95- 1.80	0.096
Fair	2.10	1.76- 2.50	< 0.000	1.60	1.23- 2.07	** < 0.001**
Bad	1.91	1.09- 3.37	0.024	1.26	0.71- 2.22	0.417
Very bad	4.47	3.81- 5.25	< 0.000	4.06	2.63- 6.26	** < 0.001**
**Isolation measures**						
Yes	Ref					
No	1.01	0.90- 1.14	0.759			
**Pandemic severity**						
Neutral	Ref					
Very serious/serious	1.04	0.88- 1.23	0.598			
Overestimated/Really overestimated	1.23	0.74- 2.05	0.418			
**Degree of confidence in the government to manage the pandemic**						
Neutral	Ref					
Much trust/some trust	0.80	0.62- 1.02	0.078			
Some distrust/distrust	1.07	0.86- 1.33	0.497			
**Previous mental health disorder**						
No	Ref			Ref		
Yes	1.46	1.13- 1.90	0.004	1.02	0.80- 1.31	0.819
**Family member with COVID-19**						
No	Ref			Ref		
Yes	1.14	1.00- 1.30	0.040	1.06	0.95- 1.17	0.253
**Family member deceased due to COVID-19**						
No	Ref					
Yes	1.16	0.95- 1.42	0.124			
**Search for mental health support**						
No	Ref			Ref		
Yes	1.49	1.23- 1.82	< 0.001	1.42	1.25- 1.62	** < 0.001**
**Loss of employment**						
Did not work	Ref			Ref		
Did not lose their job	0.87	0.70- 1.07	0.205	1.10	0.89- 1.34	0.359
Lost their job	1.71	1.49- 1.96	< 0.001	1.82	1.62- 2.04	** < 0.001**
**Anxiety symptoms**						
No	Ref					
Mild	1.39	1.03- 1.86	0.026			
Moderate	1.92	1.36- 2.70	< 0.000			
Severe	1.77	1.27- 2.46	0.001			
**Depressive symptoms**						
Minimum	Ref			Ref		
Mild	1.45	1.06- 1.99	0.018	1.24	0.91- 1–69	0.167
Moderate	1.71	1.57- 1.86	< 0.000	1.52	1.26- 1.83	** < 0.001**
Moderate to severe	2.15	1.81- 2.56	< 0.000	1.58	1.11- 2.27	**0.011**
Severe	1.85	1.40- 2.44	< 0.000	1.16	0.63- 2.07	0.603
**Post-traumatic stress disorder**						
No	Ref			Ref		
Yes	1.60	1.32- 1.95	< 0.000	1.10	0.86- 1.40	0.417
**Insomnia**	1.04	1.03- 1.05	< 0.001			
**Hypersomnia**	1.06	1.04- 1.08	< 0.001			
**Resilience**	0.99	0.99- 1.00	0.215			

^*^P-values obtained with generalized linear models using Poisson distribution, log-link function, robust variance, and places of residence as clusters

## Discussion

### Main findings

The prevalence of food insecurity was 37.1% during the initial period of the second COVID-19 epidemic wave in Peru (December 2020 and February 2021) in eight high-altitude cities of Peru. Fair and very bad perceptions of health, loss of employment, search for mental health support, and presence of depressive symptoms were associated with a higher prevalence of food insecurity.

### Prevalence of food insecurity

We found that more than a third of the participants had food insecurity (37.1%). Severe food insecurity was found in 9.3%. This is similar to what was reported in Mexico, where 9.5% had food insecurity [14]. However, it differs from a Peruvian study showing that 25.5% of people residing in the highlands had moderate to severe food insecurity. Considering the time of data collection, moderate food insecurity was 18.8% in 2015 and severe food insecurity was 4.7% in families with children under 12 years of age [7]. Conversely, in March 2020, moderate and severe food insecurity were 26.7% and 16.7%, respectively [31]. Additionally, between May and June 2020, moderate to severe food insecurity was 23.2% in the Peruvian population between 18 and 59 years old. Finally, a previous study showed that food insecurity between March and December 2020 was 24% in young adults aged 18–27 years [1]. This situation is similar to what was reported during the first wave in Brazil, in which a 47% prevalence of moderate to severe food insecurity was found [13]. These results differ from those found in Chile showing that food insecurity prevalence was 0.5%. In Bangladesh, the prevalence of severe food insecurity was higher, according to what was reported (28.3%); the same HFIAS scale was used, but the sample was much smaller [32]. During the first wave, in several countries, several restrictions were widely adopted to stop the spread of COVID-19. These restrictions, as well as the disruption of economic activities and the huge cost of public health and social security, caused a global economic crisis that affected many aspects, such as food security [33].

Food insecurity increased alarmingly after the beginning of the pandemic. The Central Reserve Bank of Peru showed a decrease of 11.1% in GDP during the first wave of the COVID-19 pandemic. However, during the second wave, the economic situation began to stabilize because the different Peruvian ministries used preparedness and contingency plans [34]. Furthermore, the poverty rate in Peru before the pandemic was 21% and after the state of emergency began, it rose to 27%, leading almost 2 million people to poverty [35]. These data could explain why food insecurity was higher during the first months of the pandemic compared to subsequent periods.Concerning the food insecurity questions, 2.0% responded that a family member often went to bed at night hungry because there was not enough food. This differs from what was reported in Bangladesh, where only 0.5% had to sleep feeling hungry [32]. Thus, since the beginning of the pandemic, families have reduced their consumption of foods such as fruits and vegetables, which could explain this food situation. This is because of the scarcity, the increase in their price, the need to buy cheaper food, and the tendency to eat non-perishable foods [36]. In addition, some households manage economic crises by obtaining support from the government and charitable organizations [37].

### Factors associated with food insecurity

We found that participants who reported a very poor perception of health had a higher prevalence of food insecurity. In addition, those with a fair perception of health had a 60% higher prevalence of food insecurity. This is consistent with the study of Pakravan-Charvadeh et al. who reported that the number of sick members in a family increases the likelihood of food insecurity during the COVID-19 outbreak [36]. Households affected by chronic diseases are at nutritional risk, as they have difficult access to food [36]. In addition, those with chronic comorbidities limiting their mobility [38], obesity, or cardiovascular risk factors [39] reported a significantly higher frequency of food insecurity.

In this study, participants who reported having lost their jobs had an 82% higher prevalence of food insecurity. This may be because Peru experienced one of the largest reductions in its labor force in 2020. Employment records for the economically active population (EAP) registered a 13% reduction, real GDP decreased by 11.5%, and household poverty increased from 20.5% to 34% [34, 40]. This differs from what was reported in another Peruvian study on young people, where 25.0% were unemployed due to the COVID-19 context [1]. Similarly, in California, USA, a study conducted on the prevalence of food insecurity and its association with unemployment in mothers of low-income households before and after the California lockdown due to COVID-19, found a 19.3% prevalence of food insecurity before the lockdown and 14.5% after it. To note, these mothers received support from public funds [41]. In addition, in Brazil, it was reported that up to 89% felt uncertain about acquiring food or receiving more [13]. A similar situation was reported by Giacoman et al., who showed that those places with unemployment in some of their members were more likely to develop food insecurity [42].

Participants who reported having sought help for a mental health problem had a 43% higher prevalence of food insecurity. This could be explained by Sparling, who concluded that it is important to conduct studies to determine the causal relationship between the search for mental health support and food insecurity [43]. In March 2020, with the announcement of the arrival of the SARS-CoV-2 virus in Peru, most economic activities were restricted, including mental health support services. This occurred worldwide, and reports in Italy mentioned that sessions with psychiatric patients would be carried out face-to-face only in urgent cases, and follow-up would be done by telephone [44]. However, it has been observed that having food insecurity predisposes the use of mental health services [45]. It is noteworthy that despite reports related to mental health during the pandemic, the influence of mental health services on food security has not yet been researched. People suffering from mental health problems are likely to face inequity in access to health services, which have been negatively impacted by the COVID-19 pandemic. In addition, these people may receive lower economic income, which could affect their food security.

Moderate and moderate to severe depressive symptoms were found to increase the prevalence of food insecurity by 52% and 58%, respectively. It should be noted that the relationship between depression and food insecurity is likely to be bidirectional. For example, food insecurity might increase the risk of depressive symptoms, or depression could be a predictor of food insecurity. [46]. This finding was similar to what was reported by Jesson et al., as they found that participants with probable depression were more likely to have food insecurity in comparison with non-depressed participants, 18% had food insecurity and 42% had probable depression. However, this study was conducted before the COVID-19 pandemic [47]. Further research is needed to determine the influence that depression may have on the onset of food insecurity, in which reduced labor productivity may be an associated factor leading to reduced income and subsequent dietary change.

### Public health implications

The results of this study provide insight into how food insecurity affects people living in high-altitude cities. Food insecurity has increased during the COVID-19 pandemic, and this research, conducted in a particular context, supports regional and international evidence. Failure to address this problem may limit the health and quality of life of families in the long term. This information should be used, along with that from other studies, to target interventions in at-risk individuals, recognizing that food insecurity is a complex situation related to a considerable number of determinants, such as those evidenced in this study. It should be noted that our findings focus on pandemic-related situations, which can serve as a possible framework for similar experiences. Especially, mental health conditions have shown relevance in how food insecurity can be triggered by psychological distress itself.

### Limitations and strengths

The study limitations include the cross-sectional study design, which does not allow us to identify causal relationships between the study variables. In addition, the study could have a selection bias, as the data were collected online and the sampling method was non-probabilistic. In addition, the results cannot be generalized to the urban or rural Peruvian population since the sample was not representative. Among the strengths, we found the use of HFIAS as a validated instrument to measure food insecurity, in addition to questionnaires to identify mental health disorders, sleep quality, and resilience. Another strength of the study is that it has captured a broad and diverse sample of eight high-altitude cities in Peru, covering remote regions in the center of the country where socioeconomic conditions are very different.

## Conclusions

Four out of ten individuals living in high-altitude cities experienced food insecurity during the second epidemic wave of COVID-19 in Peru. A higher prevalence of food insecurity was observed in people who lost their job due to the pandemic, had a fair or very bad perception of their health, searched for mental health support, and experienced moderate or moderate to severe depressive symptoms. During the pandemic, the prevalence of food insecurity has increased in the Peruvian high-altitude population, revealing the need for preventive strategies. Identification of pandemic-related characteristics that influence food insecurity can guide interventions in at-risk individuals and reduce the long-term impact of this problem on overall health and quality of life.

## Data Availability

The datasets used and analyzed during the current study are available from the corresponding author on reasonable request.
